# Pertuzumab, trastuzumab and eribulin mesylate therapy for previously treated advanced HER2-positive breast cancer: a feasibility study with analysis of biomarkers

**DOI:** 10.18632/oncotarget.24504

**Published:** 2018-02-16

**Authors:** Yasutaka Tono, Mikiya Ishihara, Yoshihiro Miyahara, Satoshi Tamaru, Hiroyasu Oda, Yoshiki Yamashita, Isao Tawara, Hiroaki Ikeda, Hiroshi Shiku, Toshiro Mizuno, Naoyuki Katayama

**Affiliations:** ^1^ Department of Hematology and Oncology, Mie University Graduate School of Medicine, 514-8507 Mie, Japan; ^2^ Department of Medical Oncology, Mie University Hospital, 514-8507 Mie, Japan; ^3^ Department of Immuno-Gene Therapy, Mie University Graduate School of Medicine, 514-8507 Mie, Japan; ^4^ Department of Oncology, Nagasaki University Graduate School of Biomedical Sciences, 852-8523 Nagasaki, Japan

**Keywords:** breast cancer, eribulin mesylate, HER2-positive, trastuzumab, pertuzumab

## Abstract

The standard treatment for advanced human epidermal growth factor receptor 2 (HER2)-positive breast cancer is the triple combination of pertuzumab, trastuzumab and docetaxel, but some patients cannot tolerate taxane. To explore a non-taxane triple therapy, we conducted a feasibility study of pertuzumab, trastuzumab and eribulin mesylate (PTE) therapy for previously treated advanced HER2-positive breast cancer with analyses of quality of life and biomarkers. Ten patients were enrolled, two of whom had a history of docetaxel allergy. The median number of prior regimens was 3. The most common Grade 3 toxicities were leukopenia (70%) and neutropenia (70%). Grade 4 or 5 adverse events were not observed. An improving trend for the Functional Assessment of Cancer Therapy-Breast (FACT-B) score at 3 months was observed. Eight cases were included in the biomarker analysis. The peripheral CD8+ T cell/ CD4+Foxp3+ regulatory T cells (Tregs) ratio was significantly increased (*p* = 0.039). The frequency of peripheral Tregs was associated with the trastuzumab trough concentration (*p* = 0.019). In a non-clinical analysis, Eribulin mesylate significantly inhibited Ser473 Akt phosphorylation in PIK3CA wild-type cells and mutated cells. These results suggest that PTE therapy is a feasible and promising option for advanced HER2-positive breast cancer. Further investigation is warranted.

## INTRODUCTION

The triple combination regimen of pertuzumab, trastuzumab and docetaxel is increasingly common because of its beneficial effects on human epidermal growth factor receptor 2 (HER2)-positive breast cancer [1–3]. However, this triple therapy is not appropriate for patients with a history of taxane allergy or those who are refractory to taxane. Thus, other safe and efficacious regimens combining a non-taxane with pertuzumab and trastuzumab are needed.

Eribulin mesylate is a non-taxane inhibitor of microtubule dynamics of the halichondrin class of antineoplastic drugs. This drug resulted in significant and clinically meaningful improvements in overall survival compared with the physician’s treatment of choice for patients with heavily pretreated metastatic breast cancer [4]. In a phase II study, the combination of trastuzumab and eribulin mesylate as a first-line therapy exhibited a 71.2% overall response rate (ORR), 11.6 months of progression-free survival (PFS) and an acceptable safety profile for locally recurrent or metastatic HER2-positive breast cancer [5]. Another study using an eribulin mesylate and trastuzumab combination reported a similar ORR and safety profile for HER2-positive breast cancer [6]. Eribulin mesylate and trastuzumab treatment is safe and yields a promising outcome for Japanese patients with HER2-positive breast cancer [7]. These data suggest that pertuzumab, trastuzumab and eribulin mesylate (PTE) could be promising as a treatment of advanced HER2-positive breast cancer.

The mechanism underlying prolonged overall survival in breast cancer patients treated with eribulin mesylate remains unclear. It is known that the serum HER2 extracellular domain (sHER) level, PIK3CA gene mutation status and trastuzumab concentration are associated with resistance to trastuzumab [8, 9]. The peripheral regulatory T cell (Treg) frequency is associated with a poor response [10], and tumor-infiltrating Tregs were correlated with decreased survival in breast cancer [11, 12].

Based on these findings, we conducted a feasibility study of PTE chemotherapy for previously treated advanced HER2-positive breast cancer, including an analysis of quality of life (QOL) and concomitant analysis of biomarkers such as sHER levels, PIK3CA gene mutation status and circulating Treg levels.

## RESULTS

### Patient characteristics

Ten patients were enrolled from October 2013 to January 2015 (Supplementary Figure 1). The patient characteristics are presented in Table 1. The median age of the patients was 60 years (range: 35–75), and the median follow-up time was 14.7 months (range: 6.9–26.6). Two patients had a history of docetaxel allergy. The median number of prior regimens for metastatic disease was 3 (1–10). The median number of prior chemoregimens for metastatic disease was 3 (0–5). One patient developed lung and lymph metastases one year after adjuvant trastuzumab completion and under adjuvant tamoxifen.

**Table 1:** Patient Characteristics

No. of patients, total Median age, years (range)	10 60 (35–75)	
	*N*	%
Sex, female	10	100
ECOG PS		
0	5	50
1	5	50
History of chemotherapy		
Anthracycline	5	50
Taxane^*^	10	100
Trastuzumab	10	100
(within 3 months of PTE)	8	80
Lapatinib	5	50
(within 3 months of PTE)	2	20
Histology		
Invasive ductal carcinoma	10	100
Hormone receptor and HER2 status		
ER+ PgR+ HER2+	4	40
ER+ PgR– HER2+	2	20
ER– PgR– HER2+	4	40
Median No. of prior regimens for metastatic disease (range)	3 (1–10)	
Median No. of prior chemoregimens for metastatic disease (range)	3 (0–5)	

^*^Two patients had a history of docetaxel allergy.

Abbreviations: ER, estrogen receptor; PgR, progesterone receptor.

### Dosage

The median number of PTE cycles was 6 (3–11). Eight patients reduced their eribulin mesylate doses from 1.4 mg/m^2^ to 1.1 mg/m^2^ due to adverse events (AEs) (two patients), skipped day 8 of eribulin mesylate therapy (four patients), or were treated with the physician’s treatment of choice (two patients). Five patients were administered ≤2.0 mg/body/day eribulin mesylate in the first cycle. Among these five patients, two required a dose reduction. All five patients whose eribulin mesylate dosage was >2.0 mg/body/day in the first cycle required a dose reduction. The dosages of pertuzumab and trastuzumab were not modified.

### AEs

The common (≥30%) treatment-related AEs included leukopenia, neutropenia, lymphopenia, diarrhea, hypokalemia, mucositis, dysgeusia, nausea, and skin disorder (Table 2). Grade 3 AEs included leukopenia (seven patients), neutropenia (eight patients), lymphopenia (two patients), febrile neutropenia (one patient), hypokalemia (one patient) and peripheral neuropathy (1 patient) (Table 2). Grade 4 or 5 AEs were not observed.

**Table 2:** Treatment-Related Adverse Events (*N* = 10)

	All grades (%)	Grade 3 (%)	Grade 4 (%)
Non-hematologic toxicities			
Diarrhea	7 (70)	0	0
Hypokalemia	7 (70)	1 (10)	0
Hypertension	2 (20)	2 (20)	0
ALT increased	4 (40)	0	0
γ-GTP increased	4 (40)	0	0
AST increased	3 (30)	0	0
Mucositis	3 (30)	0	0
Dysgeusia	3 (30)	0	0
Nausea	3 (30)	0	0
Skin disorder	3 (30)	0	0
Hyperkalemia	2 (20)	0	0
Vomiting	2 (20)	0	0
Febrile neutropenia	1 (10)	1 (10)	0
Peripheral neuropathy	1 (10)	1 (10)	0
ALP increased	1 (10)	0	0
Malaise	1 (10)	0	0
Appetite loss	1 (10)	0	0
Stomach pain	1 (10)	0	0
Myalgia	1 (10)	0	0
QTc interval prolonged	1 (10)	0	0
Hematologic toxicities			
Leukopenia	8 (80)	7 (70)	0
Neutropenia	8 (80)	7 (70)	0
Lymphopenia	7 (70)	2 (20)	0
Anemia	2 (20)	0	0
Platelet count decreased	1 (10)	0	0

Symptoms of cardiac failure were not observed. Left ventricular ejection fraction (LVEF) decreases below 50% were not observed. One patient had mild segmental hypokinesis. Her LVEF values at baseline and after treatment were 55% and 52%, respectively. She recovered 4 months after PTE discontinuation. One patient had a grade 2 corrected QT interval prolongation.

### QOL

**Figure 1:**
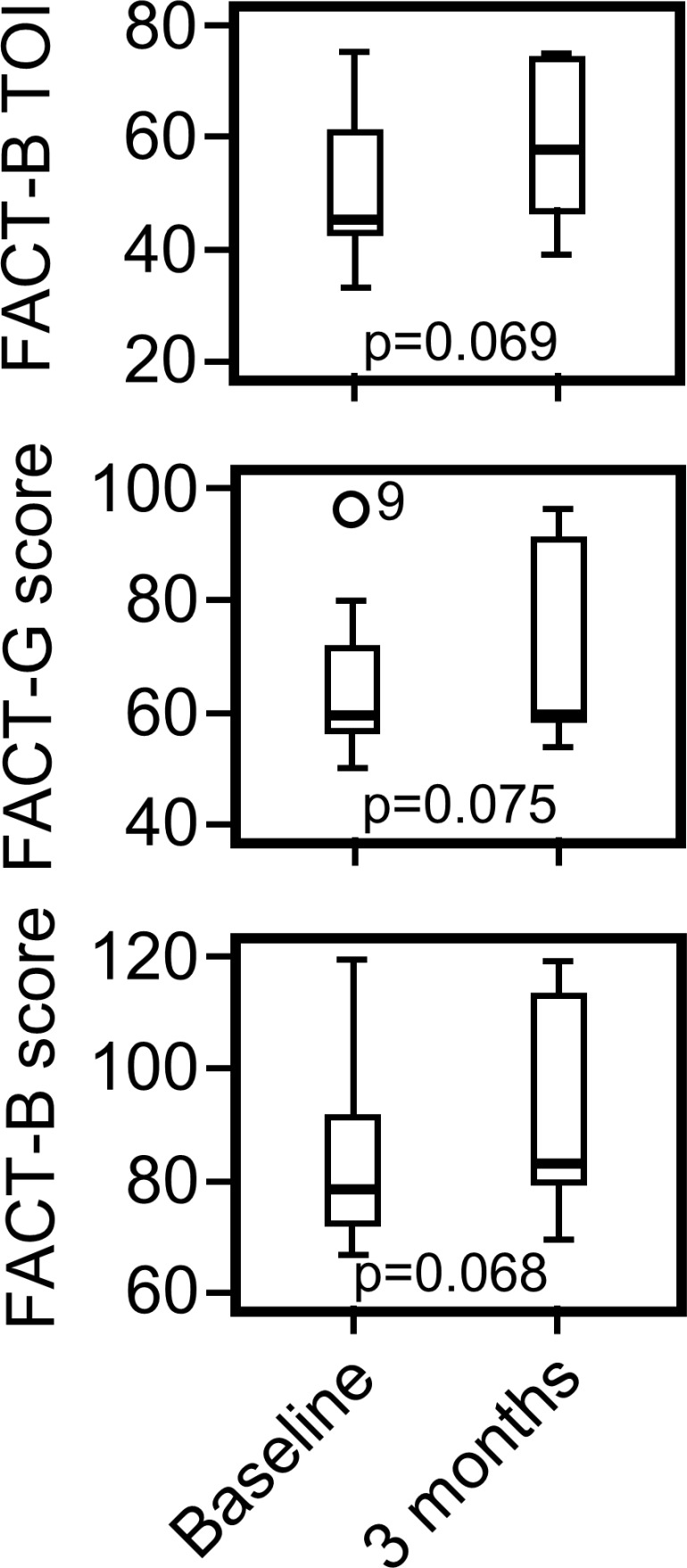
QOL assessment The FACT-B Trial Outcome Index (TOI), FACT-G Total score and FACT-B Total score at baseline and 3 months after first PTE therapy are presented.

The QOL of nine patients could be assessed. Scores at baseline and 3 months after the first PTE therapy were the Functional Assessment of Cancer Therapy-Breast (FACT-B) TOI (pre, 51.3; post, 58.3), FACT-G (pre, 65.3; post, 72.0) and FACT-B total score (pre, 84.7; post, 93.2). These scores exhibited an improving trend at 3 months, but this trend was not statistically significant (Figure 1).

### Efficacy

The median PFS was 4.8 months (95% confidence interval: 3.7–5.9). One complete response (CR), one partial response (PR) and five cases of stable disease (SD) were observed (Table 3). Two patients (one CR and one SD) stopped eribulin mesylate and received trastuzumab and pertuzumab as maintenance therapy. These patients had a PFS of more than 2 years. At 3 months, all three patients with progressive disease (PD) developed brain metastasis. Of the three PD patients, two patients had extracranial progressive lesions, and the remaining patient had a PR for extracranial disease.

**Table 3:** Response

	No. of patients (%)	95% CI (%)
CR	1 (10)	
PR	1 (10)	
SD	5 (50)	
PD	3 (30)	
Objective response rate	2 (20)	2.5–55.6
Disease control rate	7 (70)	34.8–93.3

Abbreviations: CR, complete response; PR, partial response; SD, stable disease; PD, progressive disease; CI, confidence interval.

### Flow cytometric analysis of PBMCs

**Figure 2:**
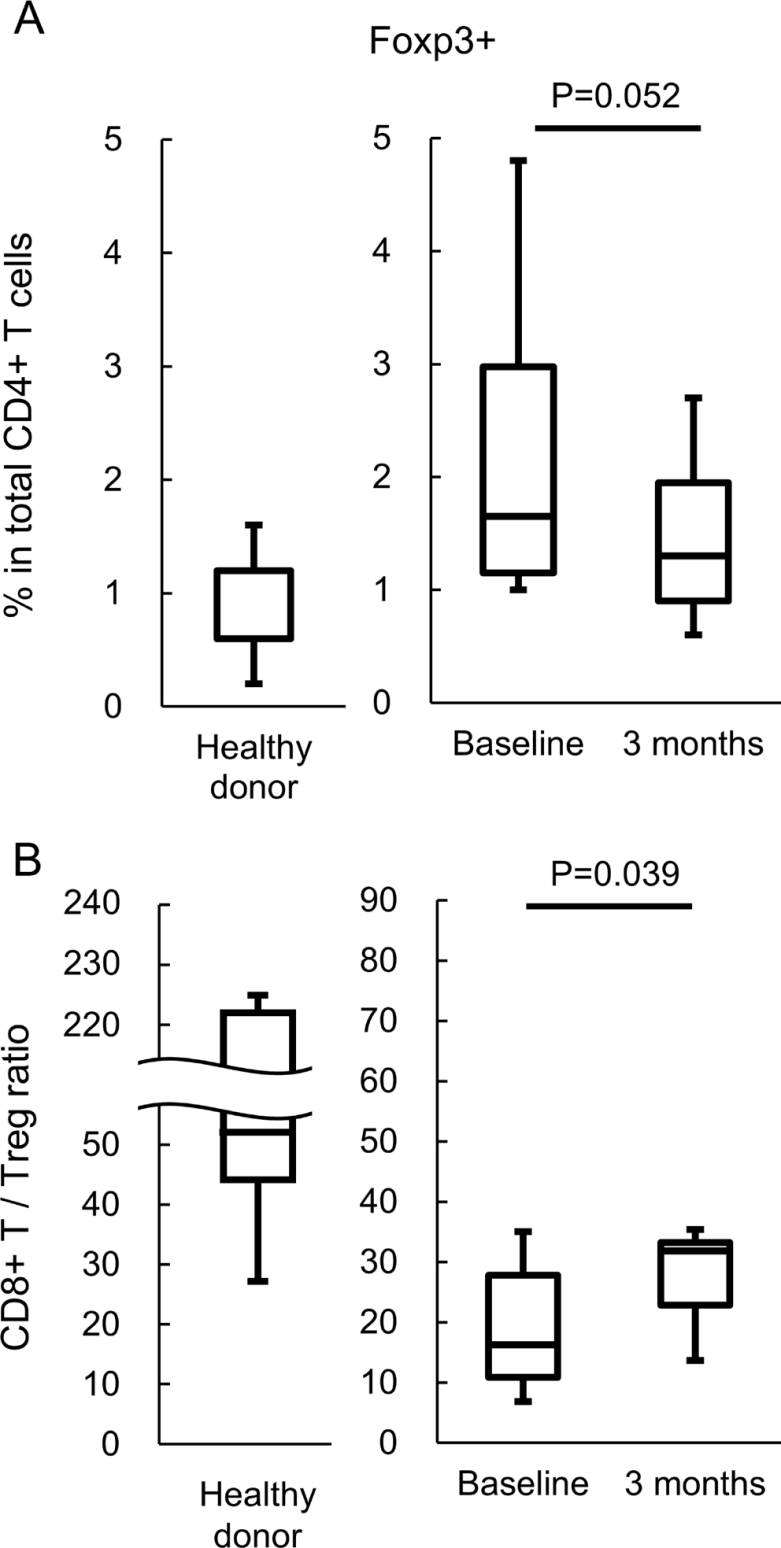
Analysis of T cell subsets The T cell subsets in peripheral blood from five healthy donors and eight patients before and 3 months after PTE therapy were assessed. (**A**) Frequency of Foxp3 expression in peripheral CD4+ T cells. (**B**) CD8+ T cells/CD4+Foxp3+ Treg ratio.

Eight cases were available for flow cytometric analysis. At 3 months, the Treg frequency exhibited a tendency to decrease (*p* = 0.052) (Figure 2A and Supplementary Table 1). In addition, the CD8+ T cell/Treg ratio was significantly increased (*p* = 0.039) (Figure 2B). The frequencies of GITR-, CTLA-4- or PD-1-positive T cells were not altered (Supplementary Figure 2). The frequencies of naïve, CM, EM or TEMRA T cells were also unchanged (data not shown).

### Correlation between trastuzumab trough concentration and Treg change

**Figure 3:**
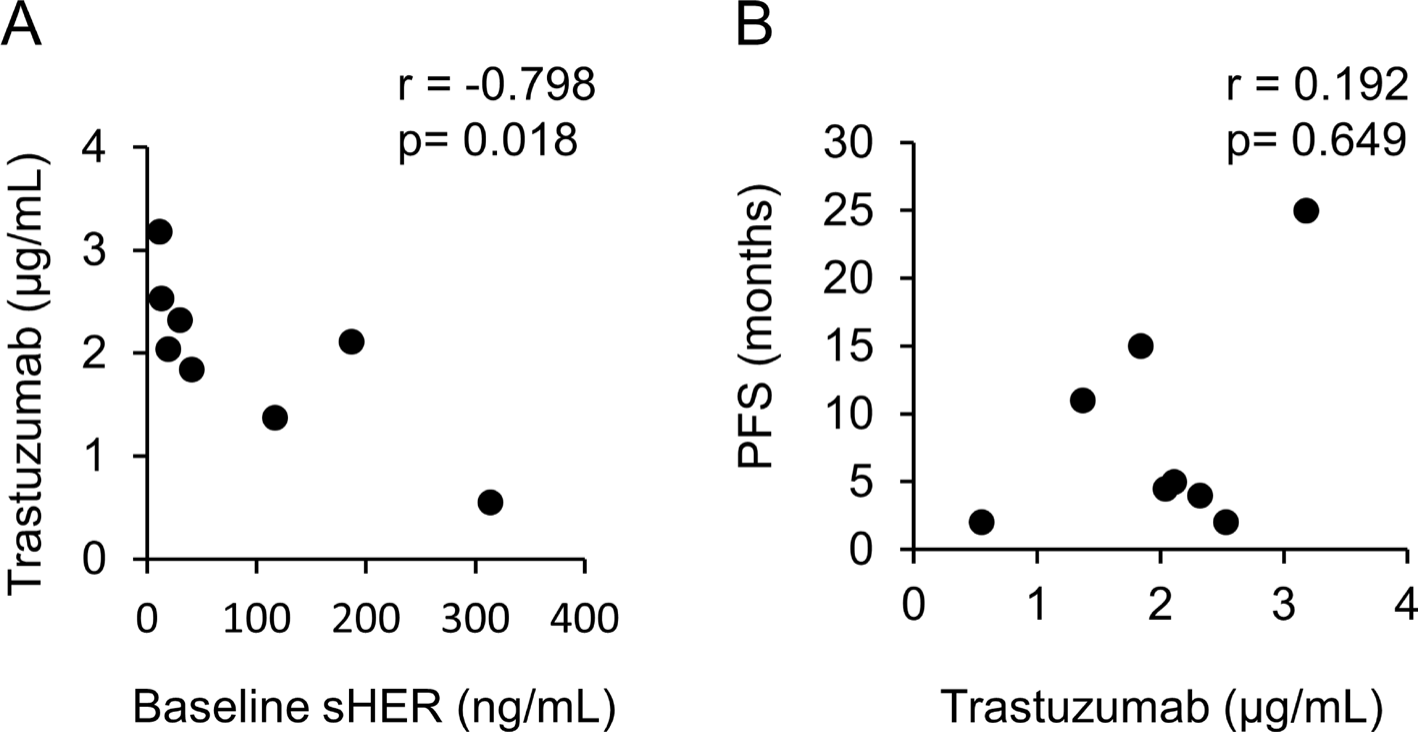
Correlation chart (**A**) Between the trastuzumab trough concentration at 3 months and sHER before treatment. (**B**) Between the trastuzumab trough concentration at 3 months and PFS.

Among the 10 enrolled patients, eight serum samples were available. The average trastuzumab concentration of two cases who received a non-trastuzumab-containing regimen immediately before PTE therapy was less than 0.1 µg/mL before treatment. However, the average trastuzumab concentration of patients who received a trastuzumab-containing regimen immediately before PTE therapy was 1.26 µg/mL (range: 0.21–2.16). The average trastuzumab trough concentration at 3 months (immediately before the next cycle) was 1.99 µg/mL (range: 0.55–3.18). With the exception of one case, the concentration of each case at 3 months was increased compared with that at baseline. The sHER values were assessed in eight cases. At baseline, two cases had normal sHER values (upper normal limit; 15.2 µg/mL), and six cases had increased sHER values. At 3 months, all six cases exhibited decreased sHER values: four cases were in the normal range; one case exhibited extreme reduction from 314.0 µg/mL to 33.4 µg/mL; and one case exhibited a moderate decrease from 187.0 µg/mL to 159.0 µg/mL. A strong negative correlation was noted between the trastuzumab trough concentration at 3 months and the baseline sHER value (*r* = –0.798) (Figure 3A). No correlation between the trastuzumab trough concentration at 3 months and PFS was noted (*r* = 0.192) (Figure 3B).

**Figure 4:**
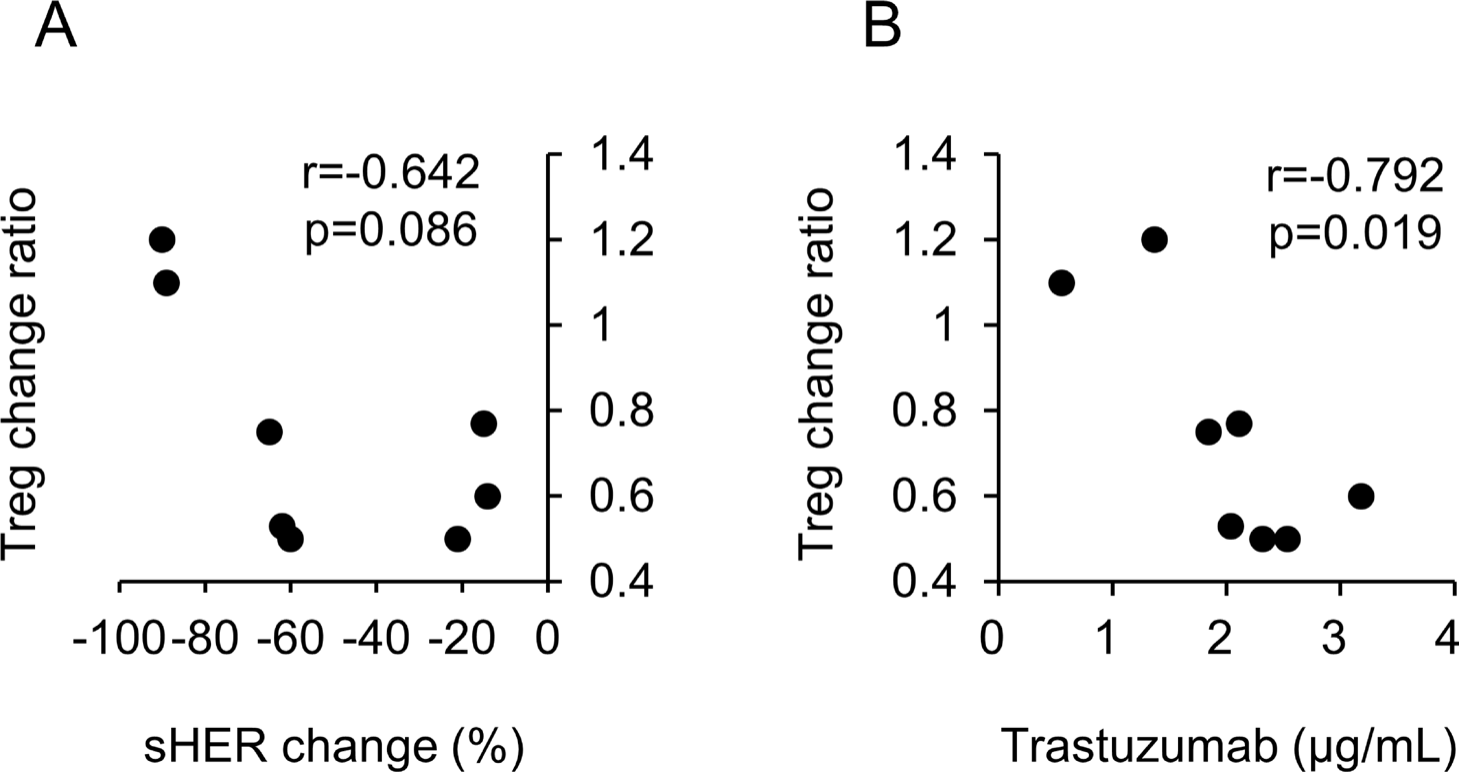
Correlation chart (**A**) Between the Treg change ratio (3 months/baseline) and sHER change {(3 months - baseline)/baseline}. (**B**) Between the Treg change ratio (3 months/baseline) and the trastuzumab trough concentration at 3 months.

The Treg change ratio and sHER change were not significantly correlated (*p* = 0.086) (Figure 4A). However, a strong negative correlation was noted between the trastuzumab trough concentration at 3 months and the Treg change (*p* = 0.019) (Figure 4B).

### Inhibition of Akt signaling by eribulin mesylate

**Figure 5:**
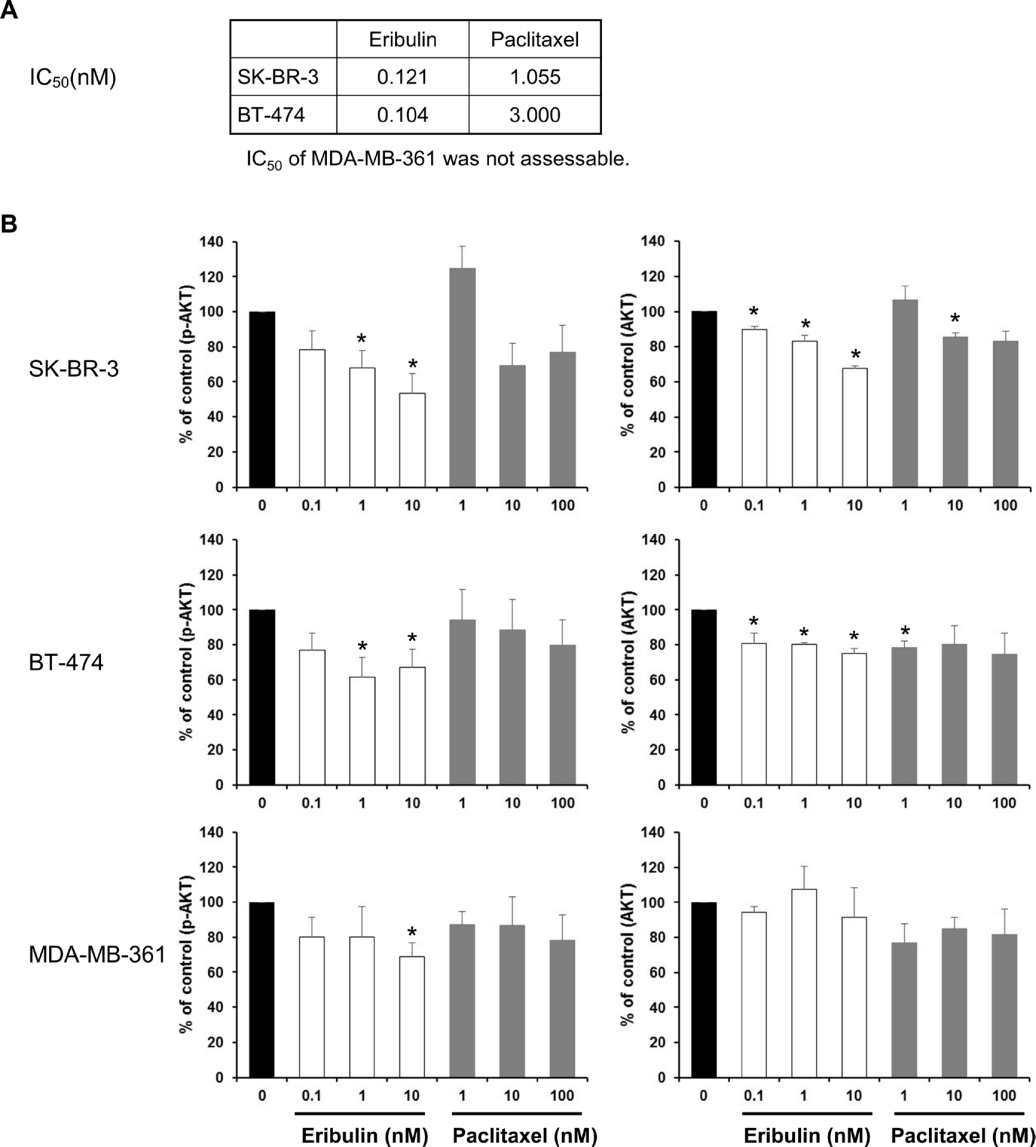
Phosphorylation of Akt (**A**) IC_50_ of SK-BR-3 (PIK3CA wild-type) and BT-474 (PIK3CA mutated-type). MDA-MB-361 (PIK3CA mutated-type) was not assessable. (**B**) Western blot assay assessing Akt phosphorylation. The cell lines were assayed after 24 hours of cultivation with eribulin mesylate or paclitaxel. The average of four experiments is presented. The data are the means + SEMs. ^*^upper 95% CI < 1 in one-sample *t*-test.

A basic research study using breast cancer cell lines was conducted as a non-clinical collaborative study between Mie University and Eisai Co., Ltd. Paclitaxel exhibited an increased 50% inhibitory concentration (IC_50_) in PIK3CA mutant cell lines compared with the PIK3CA wild-type cell line. The IC_50_ of eribulin mesylate in the PIK3CA mutant cell line BT-474 was similar to that in the PIK3CA wild-type cell line SK-BR-3 (Figure 5). The IC_50_ of eribulin mesylate in another PIK3CA mutant cell line, namely, MDA-MB-361, was not assessable due to slow cell growth. Eribulin mesylate significantly inhibited Ser473 Akt phosphorylation in PIK3CA wild-type cells and mutated cells. In contrast, paclitaxel did not exhibit significant inhibition of Ser473 Akt phosphorylation (Figure 5 and Supplementary Figure 3).

## DISCUSSION

In the present study, we attempted to evaluate the feasibility of PTE chemotherapy for previously treated advanced HER2-positive breast cancer and to analyze QOL and biomarkers such as sHER levels, PIK3CA gene mutation status and circulating Tregs. Eribulin mesylate is an attractive cytotoxic agent because it offers overall survival benefits for previously treated patients with breast cancer [4]. The overall survival benefit of eribulin mesylate was reproduced in patients with advanced sarcoma [13]. Pertuzumab is a humanized anti-HER2 monoclonal antibody [14, 15] and acts as a complementary drug of trastuzumab [16]. In the CLEOPTRA trial, the addition of pertuzumab to trastuzumab and docetaxel resulted in superior overall survival [3]. These data suggest that PTE is a promising regimen for advanced HER2-positive breast cancer. In our study, manageable tolerability was found for PTE therapy (Table 2), and the QOL (Figure 1) of patients with advanced HER2-positive breast cancer was maintained. The most common grade 3 AEs were leukopenia and neutropenia, which were reported as frequent severe AEs in previous studies of eribulin monotherapy or in combination with trastuzumab [4–7]. A dose reduction of eribulin mesylate was needed due to neutropenia in our study, and the need for dose reduction might be influenced by prior chemotherapy. When PTE therapy is administered to heavily pretreated HER2-positive breast cancer patients, 1.1 mg/m^2^ or ≤2.0 mg/body eribulin mesylate might be a reasonable dosage. The common (≥30%) non-hematologic AEs were diarrhea, hypokalemia, mucositis, dysgeusia, nausea, and skin disorder. The incidence of diarrhea in PTE therapy was higher than that of eribulin mesylate monotherapy but comparable with that reported in the CLEOPATRA study, suggesting that diarrhea is not enhanced by eribulin mesylate. Although the sample size of our study is too small to assess efficacy, PTE therapy had one CR, one PR and five SD (Table 3), and two patients who received trastuzumab and pertuzumab as maintenance therapy had a PFS of more than 2 years. Araki *et al.* recently reported a phase II clinical study of the combination therapy of pertuzumab, trastuzumab and eribulin mesylate [17]. In their trial, the ORR was 34.8%, and the PFS was 42.6 weeks. Thus, PTE therapy might be an alternative for HER2-positive breast cancer patients who are not candidates for taxane treatment. A phase III clinical study comparing pertuzumab, trastuzumab and eribulin mesylate combination therapy with pertuzumab, trastuzumab and paclitaxel or docetaxel conducted by the Japan Breast Cancer Research Group is ongoing (UMIN000027938) in Japan. This clinical trial will answer whether the PTE therapy serves as an alternative to paclitaxel or docetaxel, pertuzumab and trastuzumab.

The majority of HER2-positive metastatic breast cancer patients who achieve an initial response to trastuzumab develop resistance within 1 year [18]. Elucidation of the mechanisms of trastuzumab resistance is needed to improve the survival of HER2-positive breast cancer patients. One of the mechanisms of resistance to trastuzumab is an insufficient trastuzumab concentration [9]. Zabrecky *et al.* reported that sHER competes against trastuzumab in binding to a HER2-positive breast cancer cell line *in vitro* [19]. A previous report demonstrated that high sHER levels correlate with worse prognosis in patients receiving trastuzumab. Pertuzumab binds to HER2 extracellular domain II, which might reduce the sHER levels and increase both the serum trastuzumab concentration and trastuzumab binding to HER2-positive cancer cells. However, a high sHER level was identified as a factor for poor prognosis in the CLEOPATRA trial [20]. Thus, we hypothesized that sHER competes with trastuzumab for binding to HER2-positive cancer cells under pertuzumab and trastuzumab therapy. As shown in Figure 3A, a strong relationship was noted between the baseline sHER and the serum trastuzumab trough concentration at 3 months. Unfortunately, no significant relationship was noted between the serum trastuzumab trough concentration at 3 months and PFS (Figure 3B). This finding might be affected by other factors, such as Fcγ receptor polymorphisms [21] and the ESR1 level [22].

PIK3CA mutation is a poor prognostic factor in HER2-positive breast cancer [23, 24], and this finding was also confirmed in the CLEOPATRA trial [20]. Eribulin mesylate inhibited Akt signaling in PIK3CA-mutated cells, and this inhibition was comparable with that observed in PIK3CA wild-type cells (Figure 5). In this study, eight patients were assessed for PIK3CA status. Four patients had wild-type PIK3CA, and four patients had a mutant type. Of the four patients with PIK3CA mutations in our study, three had SD, and one had PD. One had a PFS of more than 3 years. These findings suggested that PTE triple agent therapy might be an effective treatment for HER2-positive breast cancer patients with either wild-type or mutated PIK3CA.

We observed a significant increase in the CD8+ T cells/Tregs ratio and a trend of reduced Tregs following PTE therapy. The cause of Treg reduction is an unresolved question. Perez *et al.* reported a strong positive correlation between Treg change and sHER change during trastuzumab therapy [10]. They hypothesized that sHER was eliminated from the circulation via antigen-trastuzumab complex formation and uptake by phagocytes through Fcγ receptor binding. Reduced circulating antigen subsequently reduces the expansion of antigen-specific Tregs [25, 26]. Trastuzumab and sHER complex formation could also lead to activation, maturation, and enhanced antigen cross-presentation by antigen-presenting cells [27, 28]. In our study, the correlation between Treg change and sHER change during PTE therapy was not confirmed (Figure 4A). Given that the response rate was 20%, the sHER level would not be affected by the tumor burden. Of the eight PBMC-assessable patients, six received a trastuzumab-containing regimen immediately before PTE therapy, suggesting that Treg reduction might be induced by eribulin mesylate. Eribulin mesylate could inhibit the transforming growth factor-beta (TGF-β) signaling pathway by decreasing TGF-β and/or Smad3 phosphorylation. TGF-β induced Foxp3 gene expression in T cell receptor-challenged CD4+CD25− naïve T cells, mediating their transition toward Tregs [29]. Ueda *et al.* reported that eribulin mesylate reduced the blood TGF-β concentrations in advanced breast cancer patients [30]. Eribulin mesylate could also reduce Smad2 and Smad3 phosphorylation [31]. Smad3 and/or Smad4 are required for TGF-β-mediated induction of Foxp3 in naïve CD4+ T cells [32]. These data supported the hypothesis that eribulin mesylate suppresses Treg induction via inhibition of the TGF-β/Smad pathway. Further investigation of this hypothesis is needed. The small sample size is a limitation of the Treg analysis. Further investigations are needed to confirm the increase in the CD8+ T cells/Tregs ratio after PTE therapy.

Although Tregs were reduced during PTE therapy, we did not observe any indications of T cell activation, such as significant changes in T cell subsets (naïve, CM, EM and TEMRA) and checkpoint molecule expression (CTLA-4, GITR, and PD-1), in peripheral blood (Supplementary Figure 2 and Supplementary Table 1). Because PBMCs in our flow cytometric analysis were prepared by freeze-thawing under unstimulated conditions, the expression of checkpoint molecules might be underestimated. These data suggested that T cell activation mediated by the additions of checkpoint inhibitors to PTE therapy could enhance the anti-tumour effects. A strong negative correlation was noted between the trastuzumab trough concentration at 3 months and the Treg change (Figure 4B). Petricevic *et al.* reported that antibody-dependent cellular cytotoxicity activity was similar during trastuzumab treatment [33]. Increased trastuzumab concentrations could enhance Fcγ-mediated activation of tumour-associated macrophage cytotoxicity [34, 35] and induce tumour-specific CD8+ T cells [36]. Activated macrophages might also induce tumour-specific CD4+ helper T cells, resulting in a relative reduction in Tregs. Analysis of macrophages and T cells in the tumor microenvironment and regional lymph nodes is needed.

Although the sample size of this study was small, this is the first report of QOL and Treg analyses in advanced HER2-positive breast cancer patients who received PTE therapy, and the results showed that PTE therapy maintained the QOL of patients with advanced HER2-positive breast cancer. PTE therapy might be a feasible option for advanced HER2-positive breast cancer patients, but further investigation is warranted.

## MATERIALS AND METHODS

### Patients and treatment

This was a single-institutional, open-label feasibility study of PTE for previously treated advanced HER2-positive breast cancer. Patients with a HER2-positive status [immunohistochemistry (IHC) 3+ or fluorescence *in situ* hybridization (FISH) ≥ 2.0]; aged 20–80 years; a performance status (PS) (ECOG scale) of 0–2; an LVEF ≥ 50%; a history of one or more cytotoxic agents and anti-HER2 therapy, including neo-adjuvant and adjuvant therapy; and adequate organ functions were enrolled. Patients with a history of eribulin mesylate use during the 6 months prior to consent or symptomatic brain metastasis were excluded.

Patients were treated with pertuzumab (840 mg loading, then 420 mg, day 1), trastuzumab (8 mg/kg loading, then 6 mg/kg, day 1), and eribulin mesylate (1.4 mg/m^2^, day 1 and 8) every 3 weeks. A two-step dose reduction of eribulin mesylate (starting dose 1.4 mg/m^2^, to 1.1 mg/m^2^, then to 0.7 mg/m^2^) was performed, and this approach was based on the criteria of the Japanese user guide recommended by Eisai Inc. (http://onc.eisai.jp/halaven/halaven/). The dose of eribulin mesylate was reduced when the patients developed febrile neutropenia, grade 3–5 non-hematologic toxicity or skipped eribulin mesylate administration on day 8 due to a neutrophil count <1000/mm^3^.

The primary end point of this study was the safety of PTE therapy. Secondary end points were responses, PFS, and QOL (FACT-B). The projected sample size was 10 patients, as this was a feasibility study of a novel triple therapy for previously treated advanced HER2-positive breast cancer.

The protocol was reviewed and approved by the Institutional Review Board and conducted in accordance with the Declaration of Helsinki and applicable laws. All patients provided signed informed consent before registration [*Clinical Trial Registration Number*: UMIN000012018 (Registration Date: Oct 10, 2013)].

### Assessment

AEs were assessed according to the National Cancer Institute Common Terminology Criteria for Adverse Events version 4.0. LVEF was assessed by cardiac ultrasonography every 3 months.

QOL was assessed using the FACT-B [37] prior to treatment and 3 months after PTE therapy. Japanese FACT-B version 4 consists of 37 items that are divided into five subscales: physical well-being (PWB; seven items, 0–28 points), social/family well-being (SWB; seven items, 0–28 points), emotional well-being (EWB; six items, 0–24 points), functional well-being (FWB; seven items, 0–28 points), and a breast cancer subscale (BCS; 10 items, 0–36 points). The FACT-B Trial Outcome Index (TOI), FACT-G Total score and FACT-B Total score were calculated as follows:

- FACT-B TOI = (PWB score) + (FWB score) + (BCS score) (0–92 points)
- FACT-G Total score = (PWB score) + (SWB score) + (EWB score) + (FWB score) (0–108 points)
- FACT-B Total score = (PWB score) + (SWB score) + (EWB score) + (FWB score) + (BCS score) (0–144 points)

### High scores indicate a better QOL

Computed tomography was performed every 3 months. Responses were assessed by Response Evaluation Criteria In Solid Tumors (RECIST) v1.1.

### Sample analysis

Peripheral blood mononuclear cells (PBMCs) and serum were collected before and 3 months after the first PTE treatment (immediately before the next cycle). Separated serum was cryopreserved. We investigated the serum trastuzumab concentration, sHER value, and immune status. The serum trastuzumab concentration was measured by sandwich enzyme-linked immunosorbent assay as follows: anti-trastuzumab antibodies were coated onto immunoplates at a concentration of 0.1 µg/50 µl. The collected serum samples were diluted from 1:4,000 to 1:1,024,000. After the plates were washed, goat anti-human IgG (H + L chain) (MBL, Nagoya, Japan) and IgG-peroxidase (The Binding Site, San Diego, CA, USA) were added. After the TMB substrate (Pierce, Rockford, IL, USA) was added, the plate was assessed with a microplate reader (model 550; Bio-Rad, Hercules, CA USA). The trastuzumab concentration of each sample was measured thrice, and the average value was used for the analyses. The sHER value was determined using chemiluminescent immunoassays as described by SRL Inc. (Tokyo, Japan).

PBMCs were isolated from peripheral blood by density gradient centrifugation and stored at ≤ –80° C. The immune status was assessed by flow cytometry analysis. After thawing of frozen PBMCs, aliquots containing 0.8–9.6 × 10^5^ PBMCs were suspended in 100 μL of staining buffer (phosphate buffered saline containing 2% fetal bovine serum). Antibodies for surface markers were then added, and the plates were then incubated for 15 minutes on ice. For intracellular protein (CTLA-4 and FOXP3) staining, we used the True-Nuclear™ Transcription Factor Staining Procedure (BioLegend, San Diego, CA, USA). The following antibodies were used: CD3-Brilliant Violet 711, CD4–Alexa Fluor 700, CD8a-Brilliant Violet 785, CD45RA-FITC, FOXP3-PE, CTLA-4-PE-Cy7, GITR–APC, PD-1-Brilliant Violet 421, and CCR7-Brilliant Violet 605 (BioLegend, San Diego, CA, USA). Isotype controls included the appropriate fluorochrome-conjugated mouse IgG1/κ, IgG2a/κ, or IgG2b/κ. Stained cells were detected using an LSR II Fortessa with FACS Diva software (BD Biosciences). The data were analyzed using FlowJo software (Tree Star, Ashland, OR, USA). The Treg subset was defined as CD3+CD4+FOXP3+ cells. T cells were classified as naïve (CD45RA+CCR7+), central memory (CM; CD45RA-CCR7+), effector memory (EM; CD45RA-CCR7–), and terminally differentiated effector cells (TEMRA; CD45RA+CCR7–) [38]. Appropriate isotype controls served as the cut-off levels between positivity and negativity. A positive gate was set to include less than 0.1% of cells in each specimen with a matched isotype control.

### Cell lines

SK-BR-3, BT-474 and MDA-MB-361 breast cancer cell lines were purchased from American Type Culture Collection. SK-BR-3, BT-474 and MDA-MB-361 cells were grown in McCoy’s 5A Medium supplemented with 10% fetal bovine serum (FBS), Hybri-Care Medium supplemented with FBS and DMEM/F12 medium supplemented with FBS, respectively. In total, 100 units/ml penicillin and 100 μg/ml streptomycin were added to all culture media. The cultures were maintained at 37° C in a humidified atmosphere of 5% CO_2_ and 95% air.

### Western blot assay

Briefly, SK-BR-3, BT-474 and MDA-MB-361 cells were seeded in six-well plates or 10-cm dishes in complete culture media for overnight incubation. The cells were treated with DMSO or each concentration of eribulin mesylate or paclitaxel for 24 hours. The cells were then lysed with 1× cell lysis buffer (Cell Signaling Technology, Inc., Danvers, MA, USA) plus 1 mM PMSF or RIPA buffer containing protease inhibitor cocktail (Roche Diagnostics, Mannheim, Germany) and Halt phosphatase inhibitor cocktail (Thermo Fisher Scientific, Waltham, MA, USA). The cell lysates were cleared by centrifugation, and the protein concentration was measured using the Bio-Rad Protein Assay (Bio-Rad, Hercules, CA, USA) or BCA assay (Thermo Fisher Scientific Inc., Waltham, MA, USA). The proteins were denatured in 4× sample buffer (Thermo Fisher Scientific Inc., Waltham, MA, USA), separated by SDS-PAGE and transferred onto nitrocellulose membranes or PVDF membranes. After blocking, the membranes were incubated with anti-phospho-Akt (Ser473) (Cell Signaling Technologies), anti-Akt (Cell Signaling Technologies), and anti-β-actin antibodies (Santa Cruz or Cell Signaling Technologies) at the recommended concentration overnight. Anti-rabbit IgG (LI-COR, Inc., Lincoln, NE, USA or Santa Cruz) and anti-mouse IgG (LI-COR) were used as secondary antibodies. Immunoreactive bands were visualized with an image analyzer (LI-COR Odyssey Fc Imaging System; LI-COR, Inc., Lincoln, NE, USA or LAS 4000; Fuji Film, Tokyo, Japan).

### Statistical analysis

The data for patients who were alive or lost to follow-up were censored at the last date. The Kaplan–Meier method was used to estimate the PFS. The Wilcoxon signed-rank test was used to assess the QOL scores at baseline and 3 months after the first PTE treatment. Correlation coefficients for the serum trastuzumab concentration and the sHER value, the serum trastuzumab concentration and PFS, the serum trastuzumab concentration and Treg change ratio (3 months/baseline), and the Treg change ratio (3 months/baseline) and sHER change (3 months - baseline) ^*^100/(baseline) were calculated. The paired *t*-test was used for comparisons of the immune statuses. All analyses were performed with IBM SPSS Statistics, version 23 (IBM Japan, Ltd.).

## SUPPLEMENTARY MATERIALS FIGURES AND TABLE



## Abbreviations

AEs: adverse events
CR: complete response
FACT-B: Functional Assessment of Cancer Therapy-Breast
FBS: fetal bovine serum
FISH: fluorescence *in situ* hybridization
HER2: human epidermal growth factor receptor 2
IC_50_: 50% inhibitory concentration
IHC: immunohistochemistry
ORR: overall response rate
PBMCs: peripheral blood mononuclear cells
PD: progressive disease
PFS: progression-free survival
PR: partial response
PS: performance status
PTE: pertuzumab, trastuzumab and eribulin mesylate
QOL: quality of life
RECIST: Response Evaluation Criteria In Solid Tumors
TGF-β: transforming growth factor-beta
TOI: Trial Outcome Index
Tregs: regulatory T cells
SD: stable disease
sHER: serum HER2 extracellular domain
