# Supplementary material for: Pertuzumab, trastuzumab and eribulin mesylate therapy for previously treated advanced HER2-positive breast cancer: a feasibility study with analysis of biomarkers
Source: Oncotarget. 2018 Feb 16;9(19):14909–21. doi: 10.18632/oncotarget.24504 (PMC5871086; doi:10.18632/oncotarget.24504)
Supplement: Supplementary file 1 [file oncotarget-09-14909-s001.pdf]

Pertuzumab, trastuzumab and eribulin mesylate therapy for previously treated advanced HER2-positive breast cancer: a feasibility study with analysis of biomarkers

SUPPLEMENTARY MATERIALS

Supplementary Table 1: Cell count of T cells obtained by flow cytometry

		Peripheral blood	Flow cytometry								
		Lymphocyte (/μL)	CD3+ T cells	CD4+ T cells				CD8+ T cells			
				Total	Foxp3+	CTLA-4+	GITR+	Total	CTLA-4+	GITR+	PD-1+
PTE #2	Baseline	1750	9771	6824	70	84	483	762	3	8	4
	3 months	1840	9845	6653	38	191	614	842	20	3	12
PTE #3	Baseline	1170	10000	5071	243	389	1173	2669	333	9	13
	3 months	1630	10000	3828	90	525	877	3189	702	14	12
PTE #5	Baseline	1220	10000	3857	134	578	497	3953	787	23	23
	3 months	1410	8804	3804	101	809	1076	3182	1084	10	27
PTE #6	Baseline	3300	10000	3473	62	202	235	1688	101	6	10
	3 months	2780	10000	5521	50	67	414	1771	51	5	5
PTE #7	Baseline	1640	10000	5176	78	1170	597	1161	460	2	4
	3 months	2160	10000	3305	59	564	426	1904	632	8	23
PTE #8	Baseline	790	10000	7267	89	1558	257	1588	62	4	332
	3 months	830	10000	8219	72	1642	678	982	92	17	178
PTE #9	Baseline	670	6588	3845	109	1177	366	743	92	0	221
	3 months	810	10000	6188	90	1608	455	2080	183	3	292
PTE #10	Baseline	840	10000	6771	67	732	350	2347	80	7	297
	3 months	890	10000	6386	72	1239	200	2342	144	3	474
HD #1			10000	7005	44	1476	416	1946	379	12	17
HD #2			7007	3681	45	1467	568	1223	569	19	8
HD #3			10000	3226	8	5	92	1783	7	9	1
HD #4			10000	2889	16	20	114	3525	66	10	3
HD #5			8733	3654	57	28	5.9	2944	59	9	3

Abbreviation: HD, healthy donor; PTE, pertuzumab, trastuzumab and eribulin mesylate.

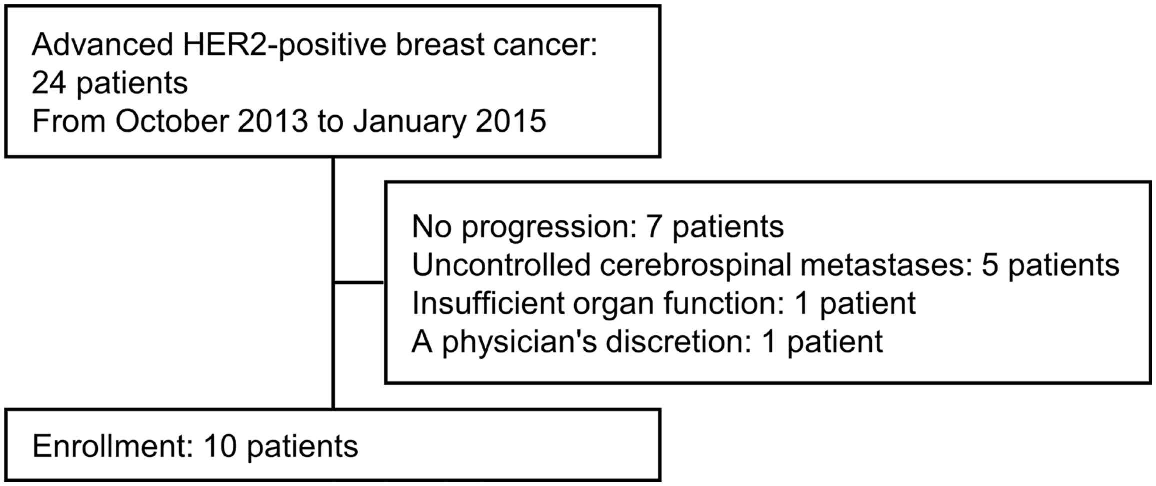

Supplementary Figure 1: Diagram of enrolment.

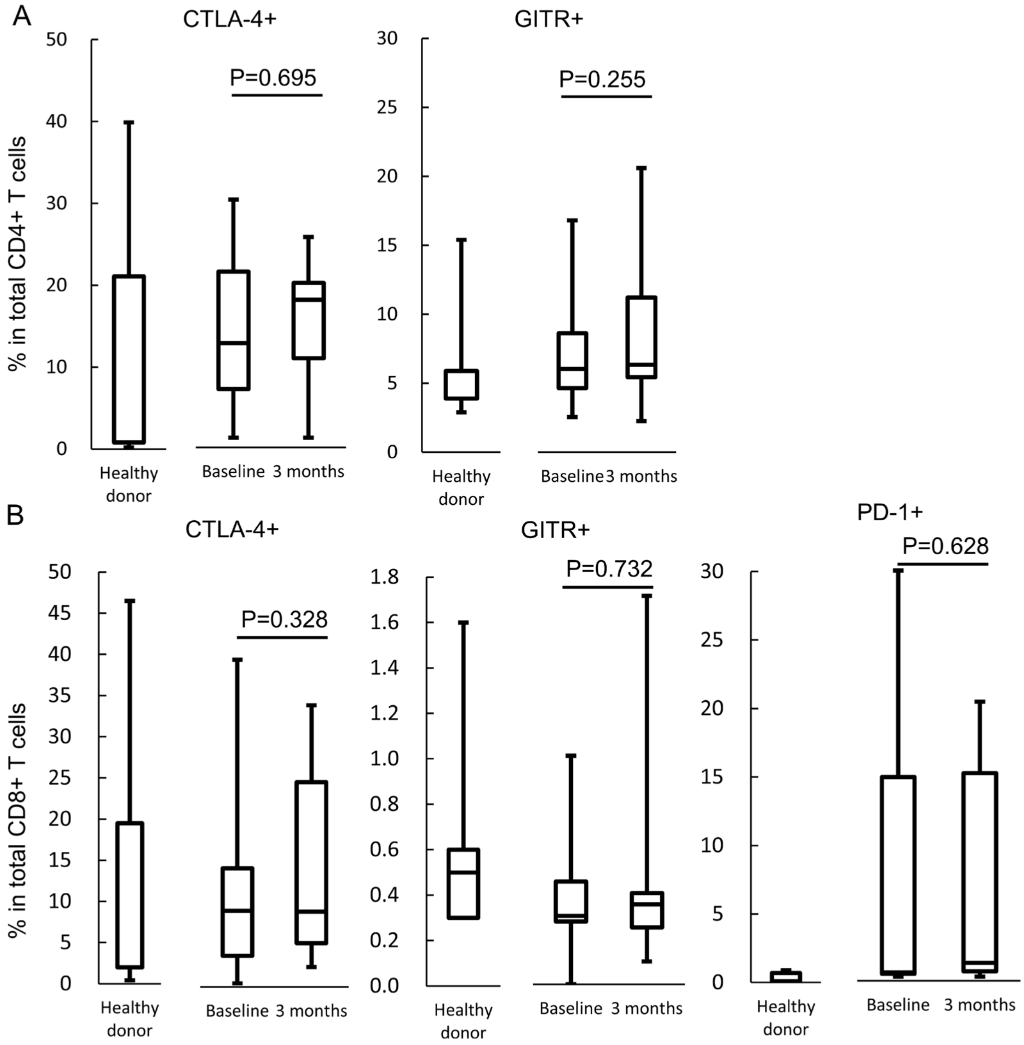

Supplementary Figure 2: Analysis of T cell subsets. The T cell subsets in peripheral blood from five healthy donors and eight patients before and 3 months after PTE therapy were assessed. (**A**) Frequency of CTLA-4 and GITR expression in peripheral CD4+ T cells. (**B**) Frequency of CTLA-4, GITR, and PD-1 expression in peripheral CD8+ T cells.

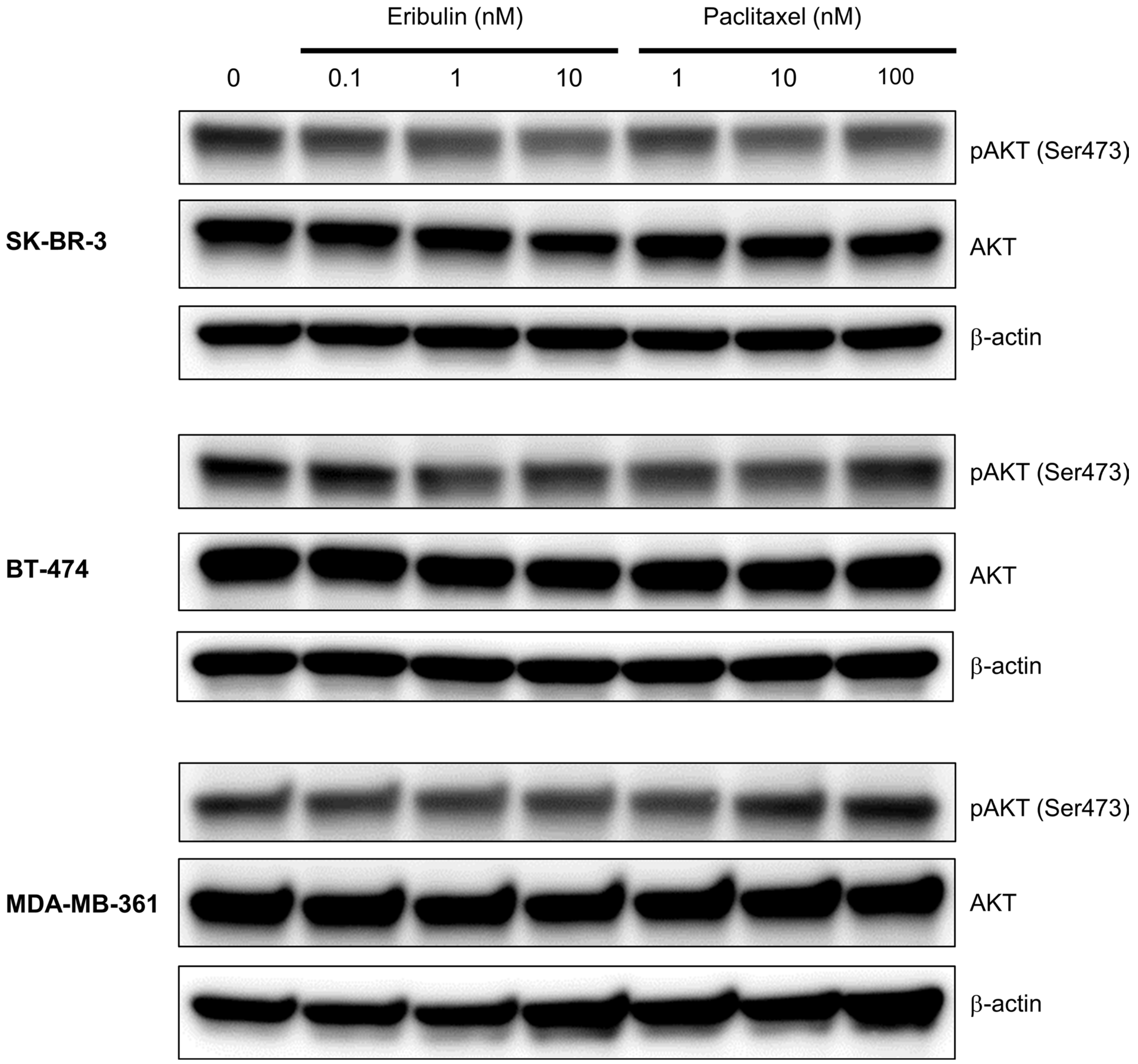

Supplementary Figure 3: Western blotting gel of Akt phosphorylation in Figure 5.
